# Bibliography

**Published:** 1920-12

**Authors:** 


### BIBLIOGRAPHY

*Crown, Bridge and Porcelain Work.* The eighth edition of this popular and pioneer text book on this subject has just appeared from the Blakeston publishing house, under the supervision of Dr. George Evans. The present edition has undergone a thorough revision, which has adapted it to the best ideas of our experts in crown and bridge work to date. The substance of the editorial revision has been to conserve the fundamental principles first worked out by Dr. Evans, and at the same time he has not left out the improved methods of our other technical experts and teachers. In the past five years bridge work particularly has been severely critized and many common methods are seriously questioned by thoughtful operators. In fact so much fault has been found with all bridge work that we do not always know where safe methods are admissible. It is therefore a matter of congratulation that so conservative and capable a writer as Dr. Evans has assumed this important task. His judgment will tend to clear up some of our questionable pronlems and assure the present as well as the future of this phase of our technical art. We are not yet ready to abandon crown and bridge work in technical dentistry, at least not until we have a competent substitute, which we don't see at present.

The author has very consistently made it clear that he will still advocate devitalizing teeth and recommend fixed bridges, when physiologic and sanitary restorations are practicable, and he gives considerable attention to the technique of both the fixed and removable bridge. We were forced to accept these methods until some better ones were devised. Dr. Evans seems to believe that it is practicable to secure adequate bridge service without pathologic results.

It is impracticable to criticize in detail this book if we were disposed to do so, and we will only say that Dr. Evans has done his work with the same conscientious devotion to his profession that has always characterized his efforts. We cordially endorse the effort he is making to remove some of the stigma that has attached itself to a most useful branch of our art, because some careless and ignorant blunderers have endeavored to prostitute a righteous cause for mercenary motives.

We sincerely believe crowns, and both fixed and removable bridges, have a most important function, and when the profession is able to adapt them properly we shall approve them because they meet our sanitary obligations.

---

*Fractures and Dislocations of the Jaws.* Under this title Dr. Chalmer J. Lyons, Professor of Dental Surgery of the University of Michigan, has contributed a number of articles in the current professional magazines on the surgical procedures of fractures of the jaws. These articles are intended to present some of the modern methods of treatment of this class of cases as they are met in the regular hospital clinics and such as resulted in the late war.

Dr. Lyons has compiled these essays and now presents them in book form for his students and members of the profession who are interested in that kind of work. The matter is valuable because it puts into accessible form much new and useful information that could not elsewhere be found. The material covers a wide field, which could not be discussed in detail or with the comprehensiveness of an extended treatise. The historic and classical introductory makes an admirable statement with which to more formally classify the material, and to present it to students and practitioners who would care to study and preserve the material for record and reference in the future. In fact we don't see why a text book for the student's use, with necessary compilations of the subject matter, would not be an admirable form for a modern and interesting treatise or handbook on the subject of strictly dental surgery. Our present books on this subject are too much elaborated either in case reports or in the technical complications, or they spend an excessive proportion of time in the details of the sciences involved.

This book has the excellent quality of interest for the student and the reader, and it should be popular, because it has a claim for originality and a message of its own. The book is published by the Ransom & Randolph Co. of Toledo, O. It is well printed and admirably typed and illustrated.

